# Translational CNS Steady-State Drug Disposition Model in Rats, Monkeys, and Humans for Quantitative Prediction of Brain-to-Plasma and Cerebrospinal Fluid-to-Plasma Unbound Concentration Ratios

**DOI:** 10.1208/s12248-021-00609-6

**Published:** 2021-06-03

**Authors:** Sho Sato, Kota Matsumiya, Kimio Tohyama, Yohei Kosugi

**Affiliations:** Global DMPK, Preclinical and Translational Sciences, Research, Takeda Pharmaceutical Company Limited, Shonan Health Innovation Park (iPark), 26-1, Muraoka-Higashi 2-Chome, Fujisawa, Kanagawa 251-8555 Japan

**Keywords:** BCRP, blood-brain barrier, K_p,uu,brain_, K_p,uu,CSF_, MDR1

## Abstract

Capturing unbound drug exposure in the brain is crucial to evaluate pharmacological effects for drugs acting on the central nervous system. However, to date, there are no reports of validated prediction models to determine the brain-to-plasma unbound concentration ratio (K_p,uu,brain_) as well as the cerebrospinal fluid (CSF)-to-plasma unbound concentration ratio (K_p,uu,CSF_) between humans and other species. Here, we developed a translational CNS steady-state drug disposition model to predict K_p,uu,brain_ and K_p,uu,CSF_ across rats, monkeys, and humans by estimating the relative activity factors (RAF) for MDR1 and BCRP in addition to scaling factors (*γ* and *σ*) using the molecular weight, logD, CSF bulk flow, and *in vitro* transport activities of these transporters. In this study, 68, 26, and 28 compounds were tested in the rat, monkey, and human models, respectively. Both the predicted K_p,uu,brain_ and K_p,uu,CSF_ values were within the 3-fold range of the observed values (71, 73, and 79%; 79, 88, and 78% of the compounds, respectively), indicating successful prediction of K_p,uu,brain_ and K_p,uu,CSF_ in the three species. The overall predictivity of the RAF approach is consistent with that of the relative expression factor (REF) approach. As the established model can predict K_p,uu,brain_ and K_p,uu,CSF_ using only *in vitro* and physicochemical data, this model would help avoid ethical issues related to animal use and improve CNS drug discovery workflow.

**Supplementary Information:**

The online version contains supplementary material available at 10.1208/s12248-021-00609-6.

## Introduction

One of the greatest challenges in drug discovery for disorders of the central nervous system (CNS) is the design of molecules with sufficient CNS exposure to achieve the desired pharmacological effects (1,2). Efflux transporters—multiple drug resistance 1 (MDR1) and breast cancer resistance protein (BCRP) expressed at the blood-brain barrier (BBB)—are mainly responsible for decreased brain penetration of drugs. These transporters play a key role by actively extruding their substrates from the brain parenchyma into the blood circulating through the brain capillary endothelial cells, resulting in lower values of the unbound brain-to-plasma drug concentration ratio (K_p,uu,brain_) (3–5). Thus, it is important to carefully select drug candidates with low transporter substrate liability in *in vitro* transport assays during the early stages of drug discovery. However, some drug candidates currently undergoing clinical study are reportedly substrates for either or both of the aforementioned transporters (6). Hence, the quantitative prediction of human K_p,uu,brain_ based on its active efflux by these transporters would be helpful to accurately estimate the appropriate human dose in addition to other nonclinical evaluations of its pharmacology and safety.

Remarkable progress has been made in quantitative prediction of brain penetration in preclinical species such as mice, rats, and monkeys (summarized in Supplemental Table 1). A pharmacokinetic model was successfully constructed to quantitatively predict K_p,uu,brain_ in rodents by considering *in vitro* efflux activities of MDR1 and/or BCRP (7–10). These studies suggested that the magnitude of K_p,uu,brain_ is predominantly governed by substrate liabilities for both MDR1 and BCRP among all the drug transporters expressed at the BBB (11,12). According to proteomics analysis of MDR1 and BCRP expressed in brain microvessels, the protein levels of these transporters differed between mice, rats, monkeys, and humans, suggesting species-dependent differences in K_p,uu,brain_ values as well (13–17). Trapa *et al.* demonstrated that K_p,uu,brain_ values in mice, rats, and monkeys could be predicted using their model of K_p,uu,brain_, when the relative expression factor (REF), corresponding to the ratio of transporter protein levels, were considered across the species (15). Although they forecasted human K_p,uu,brain_ values by extrapolating protein levels from nonclinical animals, the accuracy of their prediction is yet to be confirmed with actual values of K_p,uu,brain_. Yamamoto *et al.* and Saleh *et al.* developed sophisticated neuropharmacokinetic models in rats and humans, respectively, that predict drug concentration-time profiles within the brain by utilizing multiple physiological parameters and considering species differences (18,19). As one of the procedures to estimate MDR1- and/or BCRP-mediated active transport of drugs, which determines K_p,uu,brain_, for rat-to-human translation in their model, interspecies ratio of their protein expression level was used with assumption. Therefore, confirmation of REF-based K_p,uu,brain_ prediction across species including humans is necessary to validate this assumption (19,20).

Only a few studies measuring K_p,uu,brain_ in humans exist and will remain limited because of ethical concerns (Supplemental Table 2). Therefore, focus has increasingly shifted to measuring drug concentrations in the cerebrospinal fluid (CSF), which is more accessible than the brain, as a surrogate for brain penetration in humans (21,22). Interestingly, the unbound CSF-to-plasma concentration ratio (K_p,uu,CSF_) tends to be higher than the K_p,uu,brain_ value for MDR1 and/or BCRP substrates in rats and monkeys (22–25). Therefore, it is necessary to analyze and explain this gap between K_p,uu,brain_ and K_p,uu,CSF_ to accurately predict human K_p,uu,brain_ based on the observed K_p,uu,CSF_. To date, only Kodaira *et al.* proposed a rat pharmacokinetic model that enables to predict K_p,uu,CSF_ in addition to K_p,uu,brain_ (Supplemental Table 1) (7). Their model development was established by fitting four parameters, namely, relative activity factors (RAF) corresponding to the ratio of *in vitro* to *in vivo* efflux activities for MDR1 and BCRP, the ratio of surface area across the BBB to that across the blood-CSF barrier (BCSFB), and diffusion across the ependymal cell layer (7). These physiological considerations in rats would be helpful for the development of a model that could be applied across different species when translating nonclinical to clinical brain penetration data.

This study aimed to construct a translational CNS steady-state drug disposition model across three different species—rats, monkeys, and humans—for the prediction of K_p,uu,brain_ as well as K_p,uu,CSF_ and to examine the validity of scaling K_p,uu,brain_ values via RAF and REF approaches across species based on variable protein levels of MDR1 and BCRP.

## Materials and Methods

### Materials

Dapsone, mitoxantrone, pitavastatin calcium, and lamotrigine were purchased from Toronto Research Chemicals (Toronto, Canada). Etoposide, methotrexate, carbamazepine, erlotinib hydrochloride, and verapamil hydrochloride were purchased from FUJIFILM Wako Pure Chemical Corporation (Osaka, Japan). Pefloxacin mesylate was purchased from LKT Laboratories (St. Paul, MN, USA). Nelfinavir methanesulfonate hydrate was purchased from Tokyo Chemical Industry (Tokyo, Japan). Moxalactam disodium salt was purchased from Sigma Aldrich (St. Louis, MO, USA). Dantrolene sodium salt was purchased from Cayman (Ann Arbor, MI, USA). Prazosin hydrochloride was purchased from Santa Cruz Biotechnology (Dallas, TX, USA). Quinidine was purchased from AOBIOUS (Gloucester, MA, USA). All marketed drugs not listed above and internal molecules defined as TK-1 to TK-43 were prepared by Takeda Pharmaceutical Company (Fujisawa, Japan). All other reagents and solvents were of analytical grade and were commercially available.

### Animals

All the experimental protocols and procedures were approved by the Institutional Animal Care and Use Committee of the Shonan Health Innovation Park, Takeda Pharmaceutical Company (Unit Number: 001485), and all the animal experiments were performed at an animal research facility in Shonan Health Innovation Park accredited by Association for Assessment and Accreditation of Laboratory Animal Care International. Male cynomolgus monkeys (6–7 years old) were bred in Shin Nippon Biomedical Laboratories (Kagoshima, Japan). Male Sprague-Dawley rats (7–9 weeks old) were purchased from Horizon Discovery (Cambridge, UK).

### Compound Administration and Sampling of Plasma, CSF, and Brain

For antipyrine, carbamazepine, dantrolene, erlotinib, quinidine, sorafenib, and verapamil, all of the protocols used for the *in vivo* experiments to obtain K_p,uu,brain_ and K_p,uu,CSF_ values in rats have been described previously (8). The CSF samples collected from cisterna magna in rats were stored at –80°C until analysis by liquid chromatography-tandem mass spectrometry (LC-MS/MS). Before performing the LC-MS/MS analysis, these samples were thawed on ice and mixed with three volumes of acetonitrile. The acetonitrile mixtures were vortexed and centrifuged, and the supernatant was collected. After dilution with mobile phase A (a mixture of 10 mM ammonium formate and formic acid at 100:0.2 (v/v)), the supernatant was injected into the LC-MS/MS system.

Monkey experiments were conducted internally or at the Drug Safety Research Laboratories in Shin Nippon Biomedical Laboratories via consignment to Axcelead Drug Discovery Partners. Carbamazepine, dapsone, erlotinib, etoposide, lamotrigine, pefloxacin, methotrexate, nelfinavir, baclofen, atenolol, moxalactam, dantrolene, quinidine, prazosin, sorafenib, and verapamil were initially administered to monkeys in cassettes as an intravenous boluses and then as 6-h, constant-rate, intravenous infusions. Daidzein, genistein, and pitavastatin were administered to monkeys in cassettes as 6-h, constant-rate, intravenous infusions. Detailed dosage regimen for each compound is summarized in Supplemental Table 3. Internal molecules, TK-37 to TK-43, were administered in cassettes as an intravenous bolus dose at 0.1 to 0.5 mg/kg (Supplemental Table 2). Each cassette was assigned to a separate group of monkeys. The vehicle was saline or a mixture of dimethylacetamide and 1,3-butanediol (1:1, v/v). At the end of the 6-h infusion or 1 h after intravenous bolus administration, the monkeys were anesthetized, and whole blood, CSF, and whole brain were collected. Blood was collected using the anticoagulant, heparin sodium, and immediately centrifuged; the plasma was transferred to polypropylene tubes. Then, ketamine hydrochloride (50 mg/mL; 0.2 mL/kg; 10 mg/kg) was intramuscularly administered. CSF was collected after lumbar puncture using a spinal needle (22–25G) and a syringe pre-wetted with 10% CHAPS solution to prevent adsorption of compounds to all the equipment during pretreatment. CSF samples were immediately centrifuged, and the supernatant layers were transferred to polypropylene tubes. An aqueous pentobarbital sodium solution (64.8 mg/mL; 0.4 mL/kg) was intravenously administered, and the monkeys were euthanized by exsanguination. After collecting the cerebellum, hippocampus, and cerebral cortex from the whole brain, these were washed with cold saline, weighed, and homogenized immediately in 4 volumes of ice-cold 100 mmol/L sodium phosphate buffer. All samples were stored at –80°C until analysis by LC-MS/MS. Each sample (50 μL) was mixed with the working solution (the mixture of dimethyl sulfoxide and acetonitrile (2:8, v/v)), and protein was precipitated by the addition of 150 μL acetonitrile containing the internal standards (5 ng/mL alprenolol and diclofenac). After centrifugation at 5,250 rpm and 4°C for 5 min, the supernatant (150 μL) was transferred to a polypropylene 96-well plate (Corning; Corning, NY, USA) and completely evaporated under a gentle stream of N_2_. The residue was reconstituted in dilution solution (mobile phase A/B (7:3, v/v)), and the supernatant was analyzed by LC-MS/MS.

### Determination of Transcellular Transport Across Monolayers of MDR1- or BCRP-Expressing Cells

To the compounds available internally, *in vitro* efflux ratios for MDR1 and BCRP were determined by the following procedure (Supplemental Table 2). Test compound stock solutions in dimethyl sulfoxide (DMSO) were diluted in transport buffer (Hanks’ balanced salt solution (HBSS) with 10 mM HEPES, pH 7.4) to a concentration of 1 μM (DMSO < 1%) and applied to the apical or basolateral side of a cell monolayer derived from Madin-Darby canine kidney (MDCK)-MDR1 and MDCK-BCRP cells. Permeation of the test compounds from apical to basolateral (A to B) direction or B to A direction was measured in duplicate. The plate was incubated for 1 h in a CO_2_ incubator at 37±1°C with 5% CO_2_ at saturated humidity without shaking. In addition, the efflux ratio of each compound was also determined. Test and reference compounds were quantified by LC-MS/MS analysis based on the peak area ratio of analyte/internal standard.

The apparent permeability coefficient P_app_ (cm/s) was calculated by using Eq. 1:

1 $${\mathrm{P}}_{\mathrm{app}}=\frac{\mathrm{d}{\mathrm{C}}_{\mathrm{r}}}{\mathrm{d}\mathrm{t}}\times \frac{{\mathrm{V}}_{\mathrm{r}}}{\mathrm{A}\times {\mathrm{C}}_0}$$

where dC_r_/dt is the cumulative concentration of the compound in the receiver chamber as a function of time (μM/s); V_r_ is the volume of the solution in the receiver chamber (0.075 mL on the apical side, 0.25 mL on the basolateral side); A is the surface area for transport, i.e., 0.0804 cm^2^ for the area of the monolayer; and C_0_ is the initial concentration (μM) in the donor chamber.

The efflux ratio was calculated using Eq. 2:

2 $$\mathrm{Efflux}\ \mathrm{ratio}=\frac{{\mathrm{P}}_{\mathrm{app},\mathrm{B}\ \mathrm{to}\ \mathrm{A}}}{{\mathrm{P}}_{\mathrm{app},\mathrm{A}\ \mathrm{to}\ \mathrm{B}}}$$

### Determination of Unbound Fractions in Brain and Plasma of Rats and Monkeys

Of the compounds available internally, unbound fractions in both the brain and plasma of rats and monkeys were determined by the following procedure. An equilibrium dialysis apparatus was used to determine the unbound fraction in plasma and brain of rats and monkeys for each compound. Dialysis membranes with molecular cutoff of 6 to 8 kDa were used for dialysis. Plasma and 20% (w/v) brain homogenate in 100 mM sodium phosphate buffer were collected from male Sprague-Dawley wildtype rats and male cynomolgus monkeys. The plasma or the 20% (w/v) brain homogenate was spiked with test compound at 1 μM and dialyzed against an equal volume of 100-mM sodium phosphate buffer. The 96-well equilibrium dialysis apparatus was maintained at 37°C for 16–20 h. Both the plasma and the 20% (w/v) brain homogenate obtained from the apparatus were mixed with equal volumes of control buffer. The buffer obtained from the apparatus was mixed with an equal volume of either control plasma or control brain homogenate (20% w/v in 100 mM sodium phosphate buffer). The samples were mixed with three volumes of acetonitrile, vortexed, centrifuged, and stored at –80°C until LC-MS/MS analysis.

The unbound fractions in the brain (*f*_*u,brain*_) were calculated using Eq. 3.

3 $${\mathrm{f}}_{\mathrm{u},\mathrm{brain}}=\frac{1}{D\times \left(\frac{1}{{{\mathrm{f}}_{\mathrm{u},\mathrm{brain}}}^{\prime }}-1\right)+1}$$

where *D* and *f*_*u,brain*_' represent the dilution factor for the brain homogenate and the unbound fraction determined in the 20% (w/v) brain homogenate, respectively.

### Quantification of the Compounds in Biological Samples from Monkeys

Each sample (50 μL) was mixed with the working solution (the mixture of dimethyl sulfoxide and acetonitrile (2:8, v/v)), and the protein was precipitated by adding 150 μL acetonitrile containing internal standards (5 ng/mL alprenolol and diclofenac). After centrifugation at 5250 rpm and 4°C for 5 min, the supernatant (150 μL) was transferred to a polypropylene 96-well plate (Corning) and completely evaporated under a gentle stream of N_2_. The residue was reconstituted in mobile phase A/B (7:3, v/v)), and the supernatant was analyzed by LC-MS/MS. API5000 and QTRAP5500 (AB SCIEX, Framingham, MA, USA) equipped with Ultra-Fast Liquid Chromatography (Shimadzu, Kyoto, Japan) were used for determination of drug concentrations. Reversed-phase chromatography was performed using Shim-pack XR ODS (2.2 μm, 2.0×30 mm). Mobile phase A consisted of 10 mM ammonium formate with 0.2% formic acid, and mobile phase B contained acetonitrile with 0.2% formic acid. Gradient elution was performed as follows: 5% B at 0–0.10 min, 5–99% at 0.10–1.30 min, 99% at 1.30–2.00 min, 99–5% at 2.00–2.01 min, and 5% at 2.01–3.00 min. The mass spectrometer was operated in the positive ionization mode with multiple reaction monitoring (MRM). The MRM parameters (Q1 ion, Q3 ion, DP, EP, CE, and CXP) for monitoring the compounds are shown in Supplemental Table 4. Data acquisition and processing were performed using Analyst 1.6.2 (AB SCIEX, Framingham, MA, USA).

### Determination of K_p,uu,brain_ and K_p,uu,CSF_

After determination of the concentrations in the plasma (C_plasma_) and brain (C_brain_), K_p,uu,brain_ and K_p,uu,CSF_ were calculated by using Eqs. 4 and 5.

4 $${\mathrm{K}}_{\mathrm{p},\mathrm{uu},\mathrm{brain}}=\frac{{\mathrm{f}}_{\mathrm{u},\mathrm{brain}}\times {\mathrm{C}}_{\mathrm{brain}}}{{\mathrm{f}}_{\mathrm{u},\mathrm{plasma}}\times {\mathrm{C}}_{\mathrm{p}\mathrm{lasma}}}$$

5 $${\mathrm{K}}_{\mathrm{p},\mathrm{uu},\mathrm{CSF}}=\frac{{\mathrm{f}}_{\mathrm{u},\mathrm{CSF}}\times {\mathrm{C}}_{\mathrm{C}\mathrm{SF}}}{{\mathrm{f}}_{\mathrm{u},\mathrm{plasma}}\times {\mathrm{C}}_{\mathrm{p}\mathrm{lasma}}}$$

where f_u,CSF_ represents the unbound fraction of CSF, which was calculated using Eq. 6, as reported by Friden *et al.* (24).

6 $${\mathrm{f}}_{\mathrm{u},\mathrm{CSF}}=\frac{1}{1+{\mathrm{Q}}_{\mathrm{alb}}\times \left(\frac{1}{{\mathrm{f}}_{\mathrm{u},\mathrm{plasma}}}-1\right)}$$

where Q_alb_ is the ratio of albumin concentrations of CSF to plasma and was set to 0.003 for rats, 0.002 for monkeys, and 0.005 for humans (24,26).

### Literature Source of K_p,uu,brain_ and K_p,uu,CSF_ in Rats, Monkeys, and Humans

To perform correlation analyses between the observed K_p,uu,brain_ and K_p,uu,CSF_, and the model-based prediction of K_p,uu,brain_ and K_p,uu,CSF_, data from literature information were integrated into the internally obtained data for these assessments (Supplemental Table 2) (17,22–24,27–33). When only the total concentrations in plasma, CSF, and brain were available, the total concentration ratio in plasma to CSF or brain (K_p,CSF_ or K_p,brain_, respectively) was estimated, followed by calculation of K_p,uu,CSF_ or K_p,uu,brain_ based on Eqs. 4, 5, and 6 by using unbound fractions in the plasma and brain that were obtained internally.

### Prediction of K_p,uu,brain_ and K_p,uu,CSF_ in Rats, Monkeys, and Humans

The CNS steady-state drug disposition model used in this study was developed based on a model as reported by Kodaira *et al.* that succeeded in predicting both K_p,uu,brain_ and K_p,uu,CSF_ in rats (7). To translate these values from nonclinical animals to humans, it was extended to a model that allows prediction of K_p,uu,brain_ and K_p,uu,CSF_ not only in rats, but also in monkeys and humans by incorporating the physiological parameters of CL_bulkflow_, brain size, and body weight.

**Fig. 1:**
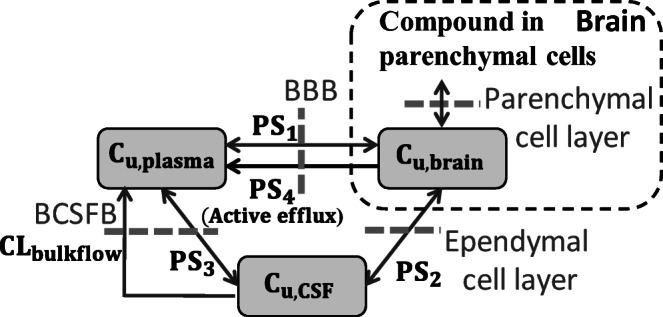
Schematic description of the developed translational CNS steady-state drug disposition model to predict both K_p,uu,brain_ and K_p,uu,CSF_ in rats, monkeys, and humans. C_u,plasma_, C_u,brain_, and C_u,CSF_ represent unbound concentrations in plasma, brain ECF, and CSF, respectively. Compound in parenchymal cells affects C_u,brain_ by substance exchange via the parenchymal cell layer. PS_1_, PS_2_, and PS_3_ represent permeability-surface area (PS) products of bidirectional passive permeability across the BBB, between the brain and CSF, and at the BCSFB, respectively. PS_4_ represents a PS product derived from active efflux transport by MDR1 (Mdr1a/1b) and BCRP (Bcrp) at BBB. CL_bulkflow_ represents CSF bulk flow rate in rats, monkeys, and humans. Unbound drug disposition among the plasma, brain, and CSF is described with relation to the PS products above and the CL_bulkflow_

This model consists of three compartments that represent unbound plasma, brain extracellular fluid (ECF), and CSF concentrations connected by passive and active efflux clearances (permeability-surface area: PS_1_, PS_2_, PS_3_, and PS_4_) and CL_bulkflow_ (Figure 1 and Table 1). PS_1_, which corresponds to influx and efflux passive clearance, was estimated by using logD (ACD Laboratories), molecular weight of compounds, and brain weight according to the literature (34). PS_2_ and PS_3_ represent influx and efflux passive clearance linked with PS_1_ divided by the fitted parameters, σ and γ,respectively. PS_4_ represents active efflux clearance linked with PS_1_ by using *in vitro* efflux ratios for MDR1 and BCRP with RAF of *α* and *β*, respectively. The CL_bulkflow_ in rats, monkeys, and humans was fixed based on the physiological values in the literature (21,35). The equation to determine PS_2_ in the present study differs from that proposed by Kodaira *et al.*; the present PS_2_ includes the contribution of PS_1_ in place of the molecular weight they used assuming bidirectional passive diffusion of unbound drug via ependymal cell layer (Table 1) (7). Since intracellular fluid (ICF) rather than ECF occupies most of the volume in the brain, the total brain drug concentration, which is determined experimentally, would primarily be represented by the brain ICF concentration. Therefore, the membrane permeation process from ICF to ECF via the parenchymal cell layer was considered to describe PS_2_ by proportional connection with PS_1_ that encompasses the BBB permeation process (Figure 1). Replacing the assumption difference on PS_2_ to the reported equation in terms of K_p,uu,brain_ and K_p,uu,CSF_ solved under steady state, K_p,uu,brain_ and K_p,uu,CSF_ can be described by PS_1_ and *in vitro* efflux ratios for MDR1 and BCRP using Eqs. 7 and 8 (7).

7 $${\mathrm{K}}_{\mathrm{p},\mathrm{uu},\mathrm{brain}}=\frac{1+\frac{\upgamma \cdotp {\mathrm{CL}}_{\mathrm{bulkflow}}}{{\mathrm{PS}}_1}+\frac{1}{\upsigma}+\frac{\upgamma}{\upsigma}}{1+\mathrm{f}\left(\mathrm{ERs}\right)+\frac{\upgamma \cdotp {\mathrm{CL}}_{\mathrm{bulkflow}}}{{\mathrm{PS}}_1}+\frac{1}{\upsigma}+\frac{\upgamma}{\upsigma}+\frac{\upgamma \cdotp {\mathrm{CL}}_{\mathrm{bulkflow}}}{\upsigma \cdotp {\mathrm{PS}}_1}+\frac{\upgamma \cdotp \mathrm{f}\left(\mathrm{ERs}\right)}{\upsigma}+\frac{\upgamma \cdotp \mathrm{f}\left(\mathrm{ERs}\right)\cdotp {\mathrm{CL}}_{\mathrm{bulkflow}}}{{\mathrm{PS}}_1}}$$

8 $${\mathrm{K}}_{\mathrm{p},\mathrm{uu},\mathrm{CSF}}=\frac{1+\mathrm{f}\left(\mathrm{ERs}\right)+\frac{1}{\upsigma}+\frac{\upgamma}{\upsigma}}{1+\mathrm{f}\left(\mathrm{ERs}\right)+\frac{\upgamma \cdotp {\mathrm{CL}}_{\mathrm{bulkflow}}}{{\mathrm{PS}}_1}+\frac{1}{\upsigma}+\frac{\upgamma}{\upsigma}+\frac{\upgamma \cdotp {\mathrm{CL}}_{\mathrm{bulkflow}}}{\upsigma \cdotp {\mathrm{PS}}_1}+\frac{\upgamma \cdotp \mathrm{f}\left(\mathrm{ERs}\right)}{\upsigma}+\frac{\upgamma \cdotp \mathrm{f}\left(\mathrm{ERs}\right)\cdotp {\mathrm{CL}}_{\mathrm{bulkflow}}}{{\mathrm{PS}}_1}}$$

**Table 1:** Parameters Used in the Translational CNS Steady-State Drug Disposition Model

Parameters	Equation	Unit	Reference	Note
PS1	$\frac{10^{\left(0.182\bullet \log {10}^{\log \frac{\mathrm{D}}{\sqrt{\mathrm{Mw}}}}-1.86\right)}}{1000}\bullet 3600\bullet \mathrm{X}$	L/h/kg	(7,24)	X:g brain/kg bodyweight
				X=rat; 7.2, rhesus monkey; 18, human; 20 g brain/kg bodyweight
PS2	$\frac{\mathrm{PS}1\bullet 60}{\upsigma \bullet 1000}$	L/h/kg	---	σ: Surface area on ependymal cell layer is proportional to that at the BBB
PS3	$\frac{\mathrm{PS}1\bullet 60}{\upgamma \bullet 1000}$	L/h/kg	(7)	γ: BCSFB is proportional to that at the BBB
PS4	{α ∙ (ER_MDR1_ − 1) + β ∙ (ER_BCRP_ − 1)} ∙ PS1	L/h/kg	(7)	α, β: scaler from *in vitro* to *in vivo* efflux activities
CL_bulkflow_	Fixed value based on physiology	L/h/kg	(18,25)	Rat (0.25 kg), 0.000528; monkey (5 kg), 0.000492
				Human (70 kg), 0.000300

Number corresponds to reference number

where f(ERs) represents the function of *in vivo* efflux activities for MDR1 and BCRP by extrapolating *in vitro* efflux ratios of these transporters using Eq. 9.

9 $$\mathrm{f}\left(\mathrm{ERs}\right)=\upalpha \cdotp \left({\mathrm{ER}}_{\mathrm{MDR}1}-1\right)+\upbeta \cdotp \left({\mathrm{ER}}_{\mathrm{BCRP}}-1\right)$$

where α represents a ratio to extrapolate the *in vitro* efflux activity of MDR1 in MDCK cells into that of MDR1 *in vivo* and β represents a ratio to extrapolate the *in vitro* efflux activity of BCRP in MDCK cells into that of BCRP *in vivo* (8).

When the value of the reciprocal of σ is small enough to be ignored, K_p,uu,brain_ can be simplified from Eq. 7 to Eq. 10, which is identical to the equation reported by the model based solely on K_p,uu,brain_ (8,15). In addition to Eq.10 at the assumption, the ratio of K_p,uu,CSF_ to K_p,uu,brain_ can be approximated as shown in Eq. 11.

10 $${\mathrm{K}}_{\mathrm{p},\mathrm{uu},\mathrm{brain}}=\frac{1}{1+\mathrm{f}\left(\mathrm{ERs}\right)}$$

11 $$\frac{{\mathrm{K}}_{\mathrm{p},\mathrm{uu},\mathrm{CSF}}}{{\mathrm{K}}_{\mathrm{p},\mathrm{uu},\mathrm{brain}}}=\frac{1+\frac{\mathrm{f}\left(\mathrm{ERs}\right)}{1+\frac{\upgamma}{\upsigma}}}{1+\frac{\upgamma \cdotp {\mathrm{CL}}_{\mathrm{bulkflow}}}{{\mathrm{PS}}_1\cdotp \left(1+\frac{\upgamma}{\upsigma}\right)}}$$

The parameters, α, β, γ, and σ, in Eq. 7 and Eq. 8 were fitted simultaneously to the logarithmic values of both the observed K_p,uu,brain_ and K_p,uu,CSF_ in rats, monkeys, and humans, respectively, for the compounds including typical substrates for MDR1 and/or BCRP using a fmincon method provided by Global Optimization Toolbox 3.5.7 equipped with Matlab R2018a (MathWorks, Natick, MA, USA).

REF for MDR1 and BCRP were estimated based on the assumption that the ratio of *in vitro* protein level is equivalent to that of *in vitro* efflux ratio between cell lines overexpressing MDR1 or BCRP as demonstrated by Feng *et al.* (36). The internally calibrated REF that Trapa *et al.* recently proposed were generated according to the procedure shown as follows (15). The correlation slope by simple linear regression of *in vitro* efflux ratios of prazosin, quinidine, metoprolol, and pitavastatin between the literature data disclosed by Trapa *et al*. and internally obtained data was determined in each MDR1- and BCRP-overexpressing cell line (Supplemental Table 2) (15). Then, REF values reported in rats, monkeys, and humans were corrected by the slope to estimate the internally calibrated REF values utilized in the present study across these three species for each MDR1 and BCRP (15).

### Correlation Analysis

Correlation of the K_p,uu_ (K_p,uu,brain_ or K_p,uu,CSF_) between the observed and predicted values was evaluated by using the average fold error (AFE) and the absolute average fold error (AAFE), both of which are indicators of accuracy and residual mean squared error (RMSE) as an index for precision. AFE, AAFE, and RMSE were calculated by using Eqs. 12–14.

12 $$\mathrm{AFE}={10}^{\frac{\sum_{k=1}^n\mathrm{Log}\frac{\mathrm{p}\mathrm{redicted}\ {\mathrm{K}}_{\mathrm{p},\mathrm{uu}}\ }{\mathrm{observed}\ {\mathrm{K}}_{\mathrm{p},\mathrm{uu}}}}{n}}$$

13 $$\mathrm{AAFE}={10}^{\frac{\sum_{k=1}^n\left|\mathrm{Log}\frac{\mathrm{p}\mathrm{redicted}\ {\mathrm{K}}_{\mathrm{p},\mathrm{uu}}\ }{\mathrm{observed}\ {\mathrm{K}}_{\mathrm{p},\mathrm{uu}}}\right|}{n}}$$

14 $$\mathrm{RMSE}=\sqrt{\frac{1}{n}\times {\sum}_{k=1}^n{\left(\mathrm{predicted}\ {\mathrm{K}}_{\mathrm{p},\mathrm{uu}}-\mathrm{observed}\ {\mathrm{K}}_{\mathrm{p},\mathrm{uu}}\right)}^2}$$

where *n* represents the size of the dataset and *k* represents *k*th data. In addition, the percentage of correlation was evaluated by using the number of compounds for which variability was within, above, and below 3-fold to the unity line. Paired *t*-tests of fold errors, absolute fold errors, and squared residual errors were used to perform statistical differences in each K_p,uu,brain_ or K_p,uu,CSF_ between the RAF and REF approaches in the CNS steady-state drug disposition model.

## Results

### Comparison of the Observed K_p,uu,brain_ and K_p,uu,CSF_ in Rats, Monkeys, and Humans

**Fig. 2:**
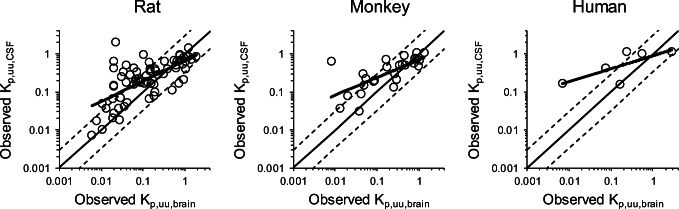
Comparison of Kp,uu,brain and Kp,uu,CSF in rats, monkeys, and humans. The observed Kp,uu,brain and Kp,uu,CSF of 70, 26, and 6 compounds were compared in rats, monkeys, and humans, respectively. The solid thin line indicates unity. Dashed lines on either side of the unity line represent three-fold factors. The solid bold line represents the linear regression curve to the logarithmic values of K_p,uu,brain_ and K_p,uu,CSF_ for the dataset

Correlation analyses between the observed values of K_p,uu,brain_ and K_p,uu,CSF_ were performed by using 70, 26, and 6 compounds from the selected set of marketed drugs and internal molecules in rats, monkeys, and humans, respectively (Figure 2). There was a tendency for K_p,uu,CSF_ to be higher than K_p,uu,brain_ in all three species according to the slopes of the logarithmic values of K_p,uu,CSF_ against K_p,uu,brain_ in the linear regression, which corresponded to 0.54, 0.47, and 0.34 in rats, monkeys, and humans, respectively. Moreover, in the correlation between K_p,uu,brain_ and K_p,uu,CSF_ in rats, compounds with K_p,uu,brain_ < 0.1 showed a wide range of K_p,uu,CSF_ values ranging from 0.01 to 1, revealing that the gap between K_p,uu,brain_ and the corresponding K_p,uu,CSF_ increases as the K_p,uu,brain_ values decreased (Figure 2).

### Predictive Performance of the Translational CNS Steady-State Drug Disposition Model in Rats, Monkeys, and Humans

**Fig. 3:**
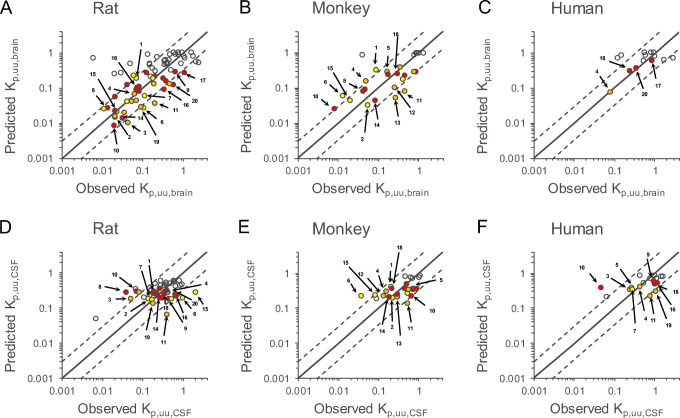
Comparison of predicted and observed values of K_p,uu,brain_ and K_p,uu,CSF_ determined using the RAF-based approach for the CNS steady-state drug disposition model in rats, monkeys, and humans. Simultaneous fitting was performed to estimate RAF of α and β and other scaling factors (γ and σ) against observed values of both K_p,uu,brain_ and K_p,uu,CSF_ in rats (**A** and **D**, respectively), monkeys (**B** and **E**, respectively), and humans (**C** and **F**, respectively). The solid black line indicates unity. Dashed lines on either side of the unity line represent three-fold factors. The red, yellow, and orange circles represent compounds with MDR1-specific, BCRP-specific, and dual substrates for both transporters, respectively, when cutoff values of *in vitro* efflux ratios in MDCK-MDR1 and MDCK-BCRP were set at 6.0 and 2.5, respectively, according to the literature from Feng *et al.* (33). The colored open circles with numbers represent marketed drugs with substrate liabilities for MDR1 and/or BCRP substrates; 1, daidzein; 2, dantrolene; 3, delavirdine; 4, erlotinib; 5, etoposide; 6, genistein; 7, indomethacin; 8, loperamide; 9, metoprolol; 10, nelfinavir; 11, pefloxacin; 12, pitavastatin; 13, prazosin; 14, quinidine; 15, sorafenib; 16, topiramate; 17, venlafaxine; 18, verapamil; 19, zidovudine; and 20, citalopram. RAF, relative activity factors

Translational CNS steady-state drug disposition models across rats, monkeys, and humans were developed by considering physiological values of CL_bulkflow_, brain size, and body weight reported in the literature (Figure 1 and Table 1) (7,21,34,35). By using these models, the RAF of α and β and other scaling factors of γ and σ were estimated by simultaneously fitting against K_p,uu,brain_ and K_p,uu,CSF_ values in rats, monkeys, and humans (Table 2). Correlations of the observed and predicted values for K_p,uu,brain_ and K_p,uu,CSF_ are shown in Figure 3. Correlation analysis was performed comprehensively based on the percentage of compounds estimated to have < 3-fold variability relative to the unity and by using statistical indices such as AFE, AAFE, and RMSE. The percentages of compounds with K_p,uu,brain_ predicted within 3-fold variability were 71, 73, and 79% in rats, monkeys, and humans, respectively (Table 2). This was fairly similar to the predicted K_p,uu,CSF_ values of 79, 88, and 78% compounds in rats, monkeys, and humans, respectively, also within 3-fold variability. The values of AFE were 1.11, 1.11, and 1.18 for K_p,uu,brain_ and 1.06, 1.01, and 0.96 for K_p,uu,CSF_ in rats, monkeys, and humans, respectively, indicating that little prediction bias was observed for both K_p,uu,brain_ and K_p,uu,CSF_ in the model across all three species. The AAFE values were 2.20, 2.24, and 1.79 for K_p,uu,brain_ and 1.94, 1.76, and 1.87 for K_p,uu,CSF_ in rats, monkeys, and humans, respectively, indicating that prediction accuracy of the model was similar across all three species (Table 2). The fitted values of RAF α, which was assumed to be the scaling factor from *in vitro* to *in vivo* activities of MDR1 expressed at the BBB, were the highest in rats at 0.395, whereas those in monkeys and humans were comparable at 0.126 and 0.096, respectively (Table 2). The fitted values of RAF β, which was assumed to be the scaling factor from *in vitro* to *in vivo* activities of BCRP expressed at the BBB, were the highest in humans at 2.054, followed by 1.368 and 0.638 in rats and monkeys, respectively (Table 2). The fitted values of γ, which was assumed to be the ratio of passive permeability through the BBB to that through the BCSFB, were comparable within 2-fold variability across species—1910, 1853, and 1468 in rats, monkeys, and humans, respectively (Table 2). The fitted values of σ, which was assumed to be the ratio of passive permeability through the BBB to that through the ependymal cell layer, were comparable within 2-fold variability across species—1054, 530, and 967 in rats, monkeys, and humans, respectively (Table 2).

**Table 2:** Predictive Performance of K_p,uu,brain_ and K_p,uu,CSF_ in Rats, Monkeys, and Humans Determined Using the RAF Approach for the CNS Steady-State Drug Disposition Model

		Rat		Monkey		Human	
		K_p,uu,brain_	K_p,uu,CSF_	K_p,uu,brain_	K_p,uu,CSF_	K_p,uu,brain_	K_p,uu,CSF_
% of predictions falling within 3-fold, below, and above	% above	15	13	19	8	14	11
	% within 3-fold	71	79	73	88	79	78
	% below	15	8	8	4	7	11
AFE		1.11	1.06	1.11	1.01	1.18	0.96
AAFE		2.20	1.94	2.24	1.76	1.79	1.87
RMSE		0.42	0.34	0.26	0.24	0.69	0.40
N		68	48	26	25	14	18
Fitted parameters (RAF)	α	0.395 ± 0.149 (38)		0.126 ± 0.075 (60)		0.096 ± 0.150 (156)	
	β	1.368 ± 0.697 (51)		0.638 ± 0.398 (62)		2.054 ± 3.341 (163)	
	γ	1910 ± 1018 (53)		1853 ± 2515 (136)		1468 ± 1774 (121)	
	σ	1054 ± 1172 (111)		530 ± 945 (178)		967 ± 1676 (173)	

*N* represents number of compounds used in the correlation analysis. Fitted parameters are shown as mean ± standard error with %CV in parenthesis. *RAF* relative activity factor

### Effects of REF Values of α and β on the Predictive Performance of the Translational CNS Steady-State Drug Disposition Model in Rats, Monkeys, and Humans

**Fig. 4:**
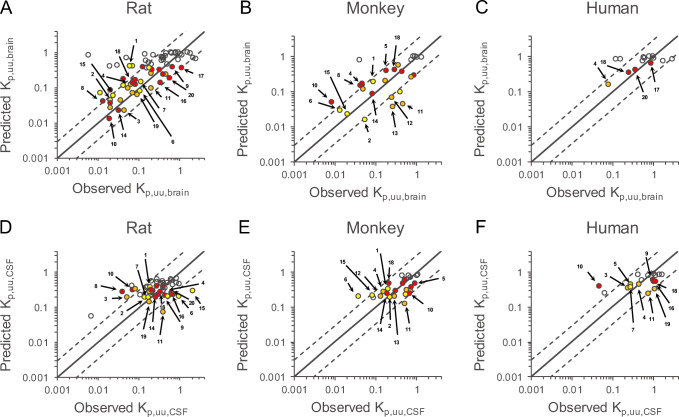
Comparison of predicted and observed values of K_p,uu,brain_ and K_p,uu,CSF_ determined using the REF-based approach for the CNS steady-state drug disposition model in rats, monkeys, and humans. Using the relative expression factor (REF) values of α and β, the values of K_p,uu,brain_ and K_p,uu,CSF_ were predicted in rats, monkeys, and humans. The comparison of the values of K_p,uu,brain_ and K_p,uu,CSF_ in rats (**A** and **D**, respectively), monkeys (**B** and **E**, respectively), and humans (**C** and **F**, respectively). The solid black line indicates unity. Dashed lines on either side of the unity line represent three-fold factors. The red, yellow, and orange circles represent compounds with MDR1-specific, BCRP-specific, and dual substrates for both transporters, respectively, when cutoff values of *in vitro* efflux ratios in MDCK-MDR1 and MDCK-BCRP were set at 6.0 and 2.5, respectively, according to the literature from Feng *et al.* (36). The colored open circles with numbers represent marketed drugs with substrate liabilities for MDR1 and/or BCRP substrates; 1, daidzein; 2, dantrolene; 3, delavirdine; 4, erlotinib; 5, etoposide; 6, genistein; 7, indomethacin; 8, loperamide; 9, metoprolol; 10, nelfinavir; 11, pefloxacin; 12, pitavastatin; 13, prazosin; 14, quinidine; 15, sorafenib; 16, topiramate; 17, venlafaxine; 18, verapamil; 19, zidovudine; 20, citalopram

To investigate the effect of REF values against the RAF values of *α* and *β* on the predictive performance of K_p,uu,brain_ and K_p,uu,CSF_ in rats, monkeys, and humans, the REF used in the present study were generated by the calibration from the reported REF using the *in vitro* efflux ratios of the scaler compounds (15). The predicted values of K_p,uu,brain_ and K_p,uu,CSF_ by using the REF values were compared with their corresponding observed values in rats, monkeys, and humans (Figure 4 and Table 3). The paired *t*-test for the fold error of the predicted against the observed values between the RAF and REF approaches showed *p*-values for K_p,uu,brain_ and K_p,uu,CSF_ were > 0.05 in rats, monkeys, and humans, with the exception for the fold error of K_p,uu,brain_ in rats showing 0.0168, indicating that there was little statistically significant difference in prediction bias between these approaches. The paired *t*-tests for both the absolute fold error and the residual error of the predicted against the observed values between the RAF and REF approaches showed *p*-values for K_p,uu,brain_ and K_p,uu,CSF_ were > 0.05 in rats, monkeys, and humans. Hence, RAF and REF did not significantly differ in terms of accuracy and precision.

**Table 3:** Predictive Performance of K_p,uu,brain_ and K_p,uu,CSF_ in Rats, Monkeys, and Humans Determined Using the REF Approach for the CNS Steady-State Drug Disposition Model

		Rat		Monkey		Human	
		K_p,uu,brain_	K_p,uu,CSF_	K_p,uu,brain_	K_p,uu,CSF_	K_p,uu,brain_	K_p,uu,CSF_
% of predictions falling within 3-fold, below, and above	% above	22	13	15	4	14	11
	% within 3-fold	75	81	65	92	79	78
	% below	3	6	19	4	7	11
AFE		1.73	1.16	1.10	1.02	1.38	1.02
AAFE		2.42	1.99	2.44	1.77	1.92	1.89
RMSE		0.42	0.35	0.26	0.23	0.66	0.39
N		68	48	26	25	14	18
Scalers used	REF of α	0.240 (1.6)		0.059 (2.1)		0.077 (1.2)	
	REF of β	0.437 (3.1)		1.248 (0.5)		0.716 (2.9)	
	γ	1910		1853		1468	
	σ	1054		530		967	

*N* represents number of compounds used in the correlation analysis. *REF* relative expression factor. Number in parenthesis represents the RAF-to-REF ratio

## Discussion

Historically, compound concentrations in CSF have been used as a surrogate for unbound brain compound concentrations because the CSF is separated by a layer of ependymal cells that have no barrier function (23,37). However, several groups reported that K_p,uu,CSF_ tended to be higher than the corresponding values of K_p,uu,brain_ in rats and monkeys (22–25). The dataset used in our study also demonstrated a similar trend in humans (Figure 2). These results suggest that the use of K_p,uu,CSF_ as a surrogate of K_p,uu,brain_ in rats, monkeys, and humans will have to be reconsidered especially under which conditions K_p,uu,CSF_ can be assumed to equal K_p,uu,brain_. While there is increasing attention to find novel procedures to predict human K_p,uu,brain_, the findings of the present study attempted to fill in this gap by establishing a modeling approach to predict K_p,uu,brain_ as well as K_p,uu,CSF_.

To examine the quantitative relationship between K_p,uu,brain_ and K_p,uu,CSF_ in each species, we expanded a previously reported pharmacokinetic model established in rats toward the three aforementioned species by considering physiological parameters (Figure 1 and Table 1) (7). Similar to Kodaira *et al*., our model consists of three compartments derived by plasma, CSF, and brain ECF with the following physiological considerations pertaining to the CSF compartment: 1) lack of tight junctions for the cells located in ependymal cell surface allows free diffusion of small molecules between the brain ECF and CSF, corresponding to PS_2_ (38,39); 2) no to little expression of MDR1 and BCRP in the plasma membrane facing blood vessels in the choroid epithelial cells suggests a lesser likelihood of active efflux of small molecules by these transporters. Rather, there would be bidirectional passive clearance between the CSF and plasma, and efflux of CSF bulk flow from CSF to plasma, corresponding to PS_3_ and CL_bulkflow_, respectively (40–42) (Figure 1).

Feng *et al.* reported a good correlation on efflux ratios between mouse and human transporter expressing MDCK cells if the functional differences of MDR1 and BCRP between mice and humans are minimal in each host cell derived by MDCK (36,43). In addition, *MDR1*-overexpressing LLC-PK1 cells showed little difference in efflux ratios between rats, monkeys, and humans (44). Therefore, we assumed that species-related differences in substrate specificity and affinity have a negligible effect on *in vitro* transport activities for MDR1 and BCRP. Hence, the species-related difference in the RAF values of α and β, estimated through model fitting by using the *in vitro* efflux ratio of human MDR1- and BCRP-overexpressing cell lines in the present study, could be assumed to be equivalent to the *in vivo* efflux activities of these transporters at the BBB (Table 2). In addition, the MDCK-MDR1 cell line was selected to determine the *in vitro* efflux activity for MDR1 in these models because we previously observed that MDCK-MDR1 had superior sensitivity in efflux ratio and correlated well with the brain disposition of different compounds (8).

In this study, the equation of PS_2_ was changed to PS_1_ multiplied by the reciprocal of σ from previously multiplying σ by the reciprocal of the square root of the molecular weight of the compound used by Kodaira *et al.* (7) (Table 1). The reasons for this change are as follows: 1) the equation adopted by Kodaira *et al.* in which the diffusion coefficient in an aqueous solution is proportional to the reciprocal of the square root of the molecular weight is only valid for molecular weights ranging from 10,000 to 147,000, but not for the small molecules of interest as in the present study (45); 2) the disposition of a drug from the brain to the CSF should consider the distribution of not only the unbound drug in the brain ECF to the CSF, but also of a drug in the parenchymal cells to the CSF via membrane penetration from the cells to the ECF space in the brain; and 3) incomprehensible scaler values were obtained in monkeys and humans when fitting the same model as Kodaira *et al.* had constructed, while these values for rats were fully reproducible (data not shown). These led us to use the new descriptive PS_2_ proportional to PS_1_ by the linkage of the reciprocal of σ in this model.

By simultaneously fitting to the observed K_p,uu,brain_ and K_p,uu,CSF_ values using the translational CNS steady-state drug disposition model developed in this study, the four scaling factors (α, β, γ, and σ) were estimated in rats, monkeys, and humans (Table 2). The γ and σ pair was a key determinant of both K_p,uu,brain_ and K_p,uu,CSF_ as shown in Eq. 7 and Eq. 8, demonstrating that the comparison of the values of γ and σ across species was expected to be meaningful to understand the gap between K_p,uu,CSF_ and K_p,uu,brain_. As shown in Table 2, the γ and σ values were comparable across the species, suggesting that the species-related differences in the ratios of passive permeabilities at BBB to BCSFB and the ependymal cell layer, respectively, were negligible among rats, monkeys, and humans. When γ was assumed to be the ratio of the polar surface area of BBB to BCSFB, the values estimated in this study were in good agreement to published data that BBB was about 3 orders of magnitude larger than BCSFB (7,40). The reciprocal values of σ were much lower than 1, leading to the approximation of K_p,uu,brain_ to Eq. 10, while the ratio of K_p,uu,CSF_ to K_p,uu,brain_ was described as shown in Eq. 11 (Table 2). These simplified equations influenced by the modeling outcome in this study suggest that the K_p,uu,brain_ was predominantly affected by the efflux activities of MDR1 and BCRP, whereas both the efflux activities and passive permeabilities of PS_1_, PS_2_, and PS_3_ were possible determinants of K_p,uu,CSF_.

A pair of the RAF values, α and β, are essential factors for K_p,uu,brain_ prediction as shown in Eqs. 7, 9, and 10. As α and β were estimated by using the same *in vitro* efflux activities for the observed values in all three species, differences in values of either α or β across the three species could arise from the species-related differences in the *in vivo* efflux activities of MDR1 or BCRP. The rank order of the RAF values of α tended to be similar to that of the corresponding REF values across the three species, with both RAF and REF showing the highest values in rats, followed by comparable values between monkeys and humans (Tables 2 and 3). The RAF-to-REF ratios of α were within 2-fold in each species, which were 1.6-, 2.1-, and 1.2-fold in rats, monkeys, and humans, respectively (Table 3). These results suggested that the RAF values of α obtained in the present study are well described according to the difference in the expression levels of MDR1 in brain microvessels across these species (13–16). On the other hand, the rank order between the RAF and REF values of β tended to be slightly different among the three species as shown in the following order: monkeys < rats < humans for the RAF values and rats < humans < monkeys for the REF values (Tables 2 and 3). Moreover, the RAF-to-REF ratios of β in rats and humans were 3.1- and 2.9-fold, respectively, and 0.5 fold in monkeys (Table 3). Further research is required before we can conclude that the large estimated %CV in humans accounted for the difference in RAF of β between animals and humans. However, these results suggested that the RAF values are unlikely to follow the BCRP expression levels reported in brain microvessels in rats and monkeys and possibly in all species (13–16). Therefore, relative functional contribution of BCRP at the BBB needs further investigation including optimization of the proteomic approach to determine protein levels of BCRP with separately quantifying the functional dimeric or larger oligomeric form of BCRP (46).

The predictivity of K_p,uu,brain_ and K_p,uu,CSF_ in rats, monkeys, and humans in the translational CNS steady-state drug disposition model was compared between the RAF and REF approaches. We found that there was no significant difference in the accuracy and precision for predicting K_p,uu,brain_ and K_p,uu,CSF_ across species. Therefore, the overall predictivity of the RAF approach is consistent with that of the REF approach. However, the predicted values of K_p,uu,brain_ were changed in response to the difference between the RAF and REF values for MDR1 and BCRP (Figures 3 and 4). Since the AFE values further from 1 and the AAFE values were unanimously larger when using REF rather than RAF in all the species examined, the RAF approach could be utilized to predict the K_p,uu,brain_ values with less prediction biases and better accuracy than the REF approach (Tables 2 and 3). The translational CNS steady-state drug disposition model by the RAF approach successfully predicted the K_p,uu,brain_ values for 71, 73, and 79% of compounds within 3-fold to the observed values in rats, monkeys, and humans, respectively (Table 2). Especially for the compounds with substrate liabilities for MDR1 and/or BCRP, the model using the RAF values showed high prediction of the magnitude of K_p,uu,brain_ as well as K_p,uu,CSF_ across species (Figure 3). Therefore, it would be informative to confirm whether the molecule of interest is BBB penetrant in a clinical setting lacking the observed K_p,uu,brain_. Although % of compounds predicted within 3-fold variability was acceptable for K_p,uu,CSF_ as well as K_p,uu,brain_, there is still room for achieving far better predictability of K_p,uu,CSF_, which was predicted within narrow dynamic range compared to K_p,uu,brain_, in our current model. To achieve this improvement, we would need to collect more data to revise our model structure through the clarification of 1) functionality and protein level of drug transporters such as MDR1, BCRP, and others at the BCSFB and *in vitro* cell lines as well for an adequate *in vitro* to *in vivo* extrapolation and 2) concentration gradient in CSF affected by sampling site difference between cisterna magna and lumbar puncture. All of these data and interpretations would be one possible key accelerator to quantitatively predict drug exposure in the CSF in prospective CNS research.

## Conclusion

In this study, we developed a translational CNS steady-state drug disposition model enabling the prediction of both K_p,uu,brain_ and K_p,uu,CSF_ among rats, monkeys, and humans by using molecular weight, logD, and *in vitro* efflux activities for MDR1 and BCRP—information that is accessible from early drug discovery stages. There was no significant difference between the RAF and REF approaches in terms of accuracy and precision for prediction of K_p,uu,brain_ and K_p,uu,CSF_. As the established model can predict K_p,uu,brain_ and K_p,uu,CSF_ quantitatively and directly using only *in vitro* and physicochemical data, this model would be beneficial in avoiding ethical issues regarding animal use and facilitate efficient CNS drug discovery workflow.

## Supplementary Information

ESM 1 (DOCX 123 kb)

## References

[1] Hammarlund-Udenaes M (2010). Active-site concentrations of chemicals - are they a better predictor of effect than plasma/organ/tissue concentrations?. Basic Clin Pharmacol Toxicol.

[2] Rankovic Z (2015). CNS drug design: balancing physicochemical properties for optimal brain exposure. J Med Chem.

[3] Schinkel AH, Smit JJ, van Tellingen O, Beijnen JH, Wagenaar E, van Deemter L (1994). Disruption of the mouse mdr1a P-glycoprotein gene leads to a deficiency in the blood-brain barrier and to increased sensitivity to drugs. Cell..

[4] Chen C, Liu X, Smith BJ (2003). Utility of Mdr1-gene deficient mice in assessing the impact of P-glycoprotein on pharmacokinetics and pharmacodynamics in drug discovery and development. Curr Drug Metab.

[5] Doyle LA, Yang W, Abruzzo LV, Krogmann T, Gao Y, Rishi AK, Ross DD (1998). A multidrug resistance transporter from human MCF-7 breast cancer cells. Proc Natl Acad Sci U S A.

[6] Wager TT, Villalobos A, Verhoest PR, Hou X, Shaffer CL (2011). Strategies to optimize the brain availability of central nervous system drug candidates. Expert Opin Drug Discovery.

[7] Kodaira H, Kusuhara H, Fuse E, Ushiki J, Sugiyama Y (2014). Quantitative investigation of the brain-to-cerebrospinal fluid unbound drug concentration ratio under steady-state conditions in rats using a pharmacokinetic model and scaling factors for active efflux transporters. Drug Metab Dispos.

[8] Sato S, Tohyama K, Kosugi Y (2020). Investigation of MDR1-overexpressing cell lines to derive a quantitative prediction approach for brain disposition using in vitro efflux activities. Eur J Pharm Sci.

[9] Adachi Y, Suzuki H, Sugiyama Y (2001). Comparative studies on in vitro methods for evaluating in vivo function of MDR1 P-glycoprotein. Pharm Res.

[10] Uchida Y, Ohtsuki S, Kamiie J, Terasaki T (2011). Blood-brain barrier (BBB) pharmacoproteomics: reconstruction of in vivo brain distribution of 11 P-glycoprotein substrates based on the BBB transporter protein concentration, in vitro intrinsic transport activity, and unbound fraction in plasma and brain in mice. J Pharmacol Exp Ther.

[11] Deo AK, Theil FP, Nicolas JM (2013). Confounding parameters in preclinical assessment of blood-brain barrier permeation: an overview with emphasis on species differences and effect of disease states. Mol Pharm.

[12] Kusuhara H, Sugiyama Y (2009). In vitro-in vivo extrapolation of transporter-mediated clearance in the liver and kidney. Drug Metab Pharmacokinet.

[13] Ito K, Uchida Y, Ohtsuki S, Aizawa S, Kawakami H, Katsukura Y, Kamiie J, Terasaki T (2011). Quantitative membrane protein expression at the blood-brain barrier of adult and younger cynomolgus monkeys. J Pharm Sci.

[14] Uchida Y, Ohtsuki S, Katsukura Y, Ikeda C, Suzuki T, Kamiie J, Terasaki T (2011). Quantitative targeted absolute proteomics of human blood-brain barrier transporters and receptors. J Neurochem.

[15] Trapa PE, Troutman MD, Lau TY, Wager TT, Maurer TS, Patel NC, West MA, Umland JP, Carlo AA, Feng B, Liras JL (2019). In vitro-in vivo extrapolation of key transporter activity at the blood-brain barrier. Drug Metab Dispos.

[16] Hoshi Y, Uchida Y, Tachikawa M, Inoue T, Ohtsuki S, Terasaki T (2013). Quantitative atlas of blood-brain barrier transporters, receptors, and tight junction proteins in rats and common marmoset. J Pharm Sci.

[17] Liu H, Dong K, Zhang W, Summerfield SG, Terstappen GC (2018). Prediction of brain:blood unbound concentration ratios in CNS drug discovery employing in silico and in vitro model systems. Drug Discov Today.

[18] Yamamoto Y, Valitalo PA, Huntjens DR, Proost JH, Vermeulen A, Krauwinkel W (2017). Predicting drug concentration-time profiles in multiple CNS compartments using a comprehensive physiologically-based pharmacokinetic model. CPT Pharmacometrics Syst Pharmacol.

[19] Saleh MAA, de Lange ECM. Impact of CNS diseases on drug delivery to brain extracellular and intracellular target sites in human: a "WHAT-IF" simulation study. Pharmaceutics. 2021;13(1). 10.3390/pharmaceutics13010095. 10.3390/pharmaceutics13010095 PMC7828633 33451111

[20] Yamamoto Y, Valitalo PA, Wong YC, Huntjens DR, Proost JH, Vermeulen A (2018). Prediction of human CNS pharmacokinetics using a physiologically-based pharmacokinetic modeling approach. Eur J Pharm Sci.

[21] de Lange EC (2013). Utility of CSF in translational neuroscience. J Pharmacokinet Pharmacodyn.

[22] Nagaya Y, Nozaki Y, Kobayashi K, Takenaka O, Nakatani Y, Kusano K, Yoshimura T, Kusuhara H (2014). Utility of cerebrospinal fluid drug concentration as a surrogate for unbound brain concentration in nonhuman primates. Drug Metab Pharmacokinet.

[23] Kodaira H, Kusuhara H, Fujita T, Ushiki J, Fuse E, Sugiyama Y (2011). Quantitative evaluation of the impact of active efflux by p-glycoprotein and breast cancer resistance protein at the blood-brain barrier on the predictability of the unbound concentrations of drugs in the brain using cerebrospinal fluid concentration as a surrogate. J Pharmacol Exp Ther.

[24] Friden M, Winiwarter S, Jerndal G, Bengtsson O, Wan H, Bredberg U (2009). Structure-brain exposure relationships in rat and human using a novel data set of unbound drug concentrations in brain interstitial and cerebrospinal fluids. J Med Chem.

[25] Nagaya Y, Katayama K, Kusuhara H, Nozaki Y (2020). Impact of P-glycoprotein-mediated active efflux on drug distribution into lumbar cerebrospinal fluid in nonhuman primates. Drug Metab Dispos.

[26] Zhang Y, Fan F, Zeng G, Zhou L, Zhang Y, Zhang J, Jiao H, Zhang T, Su D, Yang C, Wang X, Xiao K, Li H, Zhong Z (2017). Temporal analysis of blood-brain barrier disruption and cerebrospinal fluid matrix metalloproteinases in rhesus monkeys subjected to transient ischemic stroke. J Cereb Blood Flow Metab.

[27] Post RM, Uhde TW, Ballenger JC, Chatterji DC, Greene RF, Bunney WE (1983). Carbamazepine and its -10,11-epoxide metabolite in plasma and CSF. Relationship to antidepressant response. Arch Gen Psychiatry.

[28] Summerfield SG, Lucas AJ, Porter RA, Jeffrey P, Gunn RN, Read KR, Stevens AJ, Metcalf AC, Osuna MC, Kilford PJ, Passchier J, Ruffo AD (2008). Toward an improved prediction of human in vivo brain penetration. Xenobiotica.

[29] Bauer M, Karch R, Wulkersdorfer B, Philippe C, Nics L, Klebermass EM, Weber M, Poschner S, Haslacher H, Jäger W, Tournier N, Wadsak W, Hacker M, Zeitlinger M, Langer O (2019). A proof-of-concept study to inhibit ABCG2- and ABCB1-mediated efflux transport at the human blood-brain barrier. J Nucl Med.

[30] Uchida Y, Ohtsuki S, Terasaki T (2014). Pharmacoproteomics-based reconstruction of in vivo P-glycoprotein function at blood-brain barrier and brain distribution of substrate verapamil in pentylenetetrazole-kindled epilepsy, spontaneous epilepsy, and phenytoin treatment models. Drug Metab Dispos.

[31] Gatti G, Hossein J, Malena M, Cruciani M, Bassetti M (1997). Penetration of dapsone into cerebrospinal fluid of patients with AIDS. J Antimicrob Chemother.

[32] Togashi Y, Masago K, Masuda S, Mizuno T, Fukudo M, Ikemi Y, Sakamori Y, Nagai H, Kim YH, Katsura T, Mishima M (2012). Cerebrospinal fluid concentration of gefitinib and erlotinib in patients with non-small cell lung cancer. Cancer Chemother Pharmacol.

[33] Wolff M, Regnier B, Daldoss C, Nkam M, Vachon F (1984). Penetration of pefloxacin into cerebrospinal fluid of patients with meningitis. Antimicrob Agents Chemother.

[34] Davies B, Morris T (1993). Physiological parameters in laboratory animals and humans. Pharm Res.

[35] Pardridge WM (2016). CSF, blood-brain barrier, and brain drug delivery. Expert opinion on drug delivery.

[36] Feng B, West M, Patel NC, Wager T, Hou X, Johnson J, Tremaine L, Liras J (2019). Validation of human MDR1-MDCK and BCRP-MDCK cell lines to improve the prediction of brain penetration. J Pharm Sci.

[37] Liu X, Smith BJ, Chen C, Callegari E, Becker SL, Chen X, Cianfrogna J, Doran AC, Doran SD, Gibbs JP, Hosea N, Liu J, Nelson FR, Szewc MA, van Deusen J (2006). Evaluation of cerebrospinal fluid concentration and plasma free concentration as a surrogate measurement for brain free concentration. Drug Metab Dispos.

[38] Lin JH (2008). CSF as a surrogate for assessing CNS exposure: an industrial perspective. Curr Drug Metab.

[39] Liu X, Van Natta K, Yeo H, Vilenski O, Weller PE, Worboys PD (2009). Unbound drug concentration in brain homogenate and cerebral spinal fluid at steady state as a surrogate for unbound concentration in brain interstitial fluid. Drug Metab Dispos.

[40] de Lange EC (2004). Potential role of ABC transporters as a detoxification system at the blood-CSF barrier. Adv Drug Deliv Rev.

[41] Rao VV, Dahlheimer JL, Bardgett ME, Snyder AZ, Finch RA, Sartorelli AC, Piwnica-Worms D (1999). Choroid plexus epithelial expression of MDR1 P glycoprotein and multidrug resistance-associated protein contribute to the blood-cerebrospinal-fluid drug-permeability barrier. Proc Natl Acad Sci U S A.

[42] Zhuang Y, Fraga CH, Hubbard KE, Hagedorn N, Panetta JC, Waters CM, Stewart CF (2006). Topotecan central nervous system penetration is altered by a tyrosine kinase inhibitor. Cancer Res.

[43] Feng B, Mills JB, Davidson RE, Mireles RJ, Janiszewski JS, Troutman MD, de Morais SM (2008). In vitro P-glycoprotein assays to predict the in vivo interactions of P-glycoprotein with drugs in the central nervous system. Drug Metab Dispos.

[44] Takeuchi T, Yoshitomi S, Higuchi T, Ikemoto K, Niwa S, Ebihara T (2006). Establishment and characterization of the transformants stably-expressing MDR1 derived from various animal species in LLC-PK1. Pharm Res.

[45] Nugent LJ, Jain RK (1984). Extravascular diffusion in normal and neoplastic tissues. Cancer Res.

[46] Jani M, Ambrus C, Magnan R, Jakab KT, Beery E, Zolnerciks JK (2014). Structure and function of BCRP, a broad specificity transporter of xenobiotics and endobiotics. Arch Toxicol.

