# Supplementary material for: Translational CNS Steady-State Drug Disposition Model in Rats, Monkeys, and Humans for Quantitative Prediction of Brain-to-Plasma and Cerebrospinal Fluid-to-Plasma Unbound Concentration Ratios
Source: AAPS J. 2021 Jun 3;23(4):81. doi: 10.1208/s12248-021-00609-6 (PMC8175309; doi:10.1208/s12248-021-00609-6)
Supplement: Supplementary file 1 — (DOCX 123 kb) [file 12248_2021_609_MOESM1_ESM.docx]

**Supplemental Table 1 Summary of current published information relating to prediction of K_p,uu,brain_ and K_p,uu,CSF_ of substrates for MDR1 and BCRP**

|  | **K_p,uu,brain_** | | **K_p,uu,CSF_** | |
| --- | --- | --- | --- | --- |
| Substrate liability of test compounds | MDR1 | BCRP | MDR1 | BCRP |
| Mouse | (9, 10, 15) | (15) | Not reported | Not reported |
| Rat | (7, 8, 15) | (7, 8, 15) | (7) | (7) |
| Monkey | (15),* | (15) | Not reported | Not reported |
| Human | Not reported | Not reported | Not reported | Not reported |

*****Uchida Y, Wakayama K, Ohtsuki S, Chiba M, Ohe T, Ishii Y, et al. Blood-brain barrier pharmacoproteomics-based reconstruction of the *in vivo* brain distribution of P-glycoprotein substrates in cynomolgus monkeys. The Journal of Pharmacology and Experimental Therapeutics. 2014;350(3):578-88. doi:10.1124/jpet.114.214536.

**Supplemental Table 2 Summary of dataset to be used to compare K_p,uu,brain_ with K_p,uu,CSF_ and predict K_p,uu,brain_ and K_p,uu,CSF_ from physicochemical and *in vitro* parameters in rats, monkeys, and humans**

| Compound name | Figure type | | Mw | LogD | Efflux ratio | | | | K_p,uu,CSF_ | | | | | | K_p,uu,brain_ | | | | | |
| --- | --- | --- | --- | --- | --- | --- | --- | --- | --- | --- | --- | --- | --- | --- | --- | --- | --- | --- | --- | --- |
|  | a | b |  |  | MDR1 | | BCRP | | Rat | | Monkey | | Human | | Rat | | Monkey | | Human | |
| Amitriptyline | Y | N | 277.4 | 2.96 | NA |  | NA |  | 0.17 | ^*4^ | NA |  | 0.18 | ^*4^ | 0.73 | ^*4^ | NA |  | NA |  |
| Antipyrine | Y | Y | 188.2 | 0.72 | 0.9 | ^*1^ | 1.0 | ^*1^ | 0.944  0.990 | ^*1^  ^*7^ | 1.05 | ^*9^ | NA |  | 0.857  0.708 | ^*1,^  ^*7^ | 0.857 | ^*9^ | NA |  |
| Atenolol | Y | N | 266.3 | -1.85 | NA |  | 1.2 | ^*1^ | 0.036 | ^*4^ | 0.331 | ^*1^ | 0.54 | ^*4^ | 0.026 | ^*4^ | NA |  | NA |  |
| Baclofen | Y | N | 213.7 | -0.94 | NA |  | NA |  | 0.027 | ^*4^ | 0.030 | ^*1^ | 0.17 | ^*4^ | 0.02 | ^*4^ | 0.037 | ^*1^ | NA |  |
| Carbamazepine | Y | Y | 236.3 | 2.28 | 1.3 | ^*1^ | 0.9 | ^*1^ | 0.752  0.535 | ^*1^  ^*7^ | 0.979  1.19 | ^*1^  ^*9^ | 1.00 | ^*10^ | 0.80  0.39  0.771 | ^*1^  ^*3^  ^*7^ | 1.445  1.27 | ^*1^  ^*9^ | 0.55 | ^*3^ |
| Cefotaxime | Y | N | 455.5 | NA | NA |  | NA |  | NA |  | NA |  | 0.17 | ^*4^ | NA |  | NA |  | 0.007 | ^*4^ |
| Citalopram | Y | Y | 324.4 | 1.27 | 18.0 | ^*1^ | 0.6 | ^*1^ | 0.667 | ^*7^ | NA |  | NA |  | 0.623  0.494 | ^*3^  ^*7^ | NA |  | 0.347 | ^*3^ |
| Codeine | Y | N | 299.4 | NA | NA |  | NA |  | 0.54 | ^*4^ | NA |  | 0.79 | ^*4^ | 0.89 | ^*4^ | NA |  | NA |  |
| Daidzein | Y | Y | 254.2 | 2.38 | 0.3 | ^*1^ | 4.1 | ^*1^ | 0.189 | ^*7^ | 0.201 | ^*1^ | NA |  | 0.0667 | ^*7^ | 0.085 |  | NA |  |
| Dantrolene | Y | Y | 314.3 | 1.33 | 1.3 | ^*1^ | 48.0 | ^*1^ | 0.19  0.084 | ^*1^  ^*7^ | 0.220 | ^*1^ | NA |  | 0.01  0.0297 | ^*1^  ^*7^ | 0.053 | ^*1^ | NA |  |
| Dapsone | Y | Y | 248.3 | 1.08 | 1.3 | ^*1^ | 0.8 | ^*1^ | NA |  | 0.661 | ^*1^ | 1.56 | ^*11^ | NA |  | 1.025 | ^*1^ | NA |  |
| Delavirdine | Y | Y | 456.6 | 1.52 | 110 | ^*1^ | 38.0 | ^*1^ | 0.051 | ^*4^ | NA |  | 0.23 | ^*4^ | 0.043 | ^*4^ | NA |  | NA |  |
| Diazepam | Y | N | 284.7 | NA | NA |  | NA |  | 0.78  0.847 | ^*4^  ^*7^ | NA |  | 0.79 | ^*4^ | 1.07  0.805 | ^*4^  ^*7^ | 0.877 | ^*8^ | NA |  |
| Diphenhydramine | Y | Y | 255.4 | 2.34 | 1.6 | ^*1^ | 1.0 | ^*1^ | 0.39 | ^*4^ | NA |  | NA |  | 1.050 | ^*4^ | NA |  | NA |  |
| Erlotinib | Y | Y | 393.4 | 3.05 | 19.8 | ^*1^ | 5.8 | ^*1^ | 0.409 | ^*1^ | 0.151 | ^*1^ | 0.428 | ^*2^ | 0.061 | ^*1^ | 0.049 | ^*1^ | 0.076 | ^*5^ |
| Ethyl-phenylmalonamide | Y | N | 206.2 | NA | NA |  | NA |  | 1.4 | ^*4^ | NA |  | NA |  | 1.250 | ^*4^ | NA |  | NA |  |
| Etoposide | Y | Y | 588.6 | 0.66 | 6.1 | ^*1^ | 3.7 | ^*1^ | NA |  | 0.702 | ^*1^ | 0.265 |  | NA |  | 0.164 | ^*1^ | NA |  |
| Flumazenil | N | Y | 303.3 | 1.00 | 2.9 | ^*1^ | 1.2 | ^*1^ | NA |  | NA |  | NA |  | NA |  | NA |  | 1.67 | ^*3^ |
| Fluoxetine | N | Y | 309.3 | 1.75 | 2.2 | ^*1^ | 1.3 | ^*1^ | NA |  | NA |  | NA |  | 2.143 | ^*3^ | NA |  | 0.53 | ^*3^ |
| Gabapentin | Y | N | 171.2 | NA | NA |  | NA |  | 0.067 | ^*4^ | NA |  | 0.16 | ^*4^ | 0.140 | ^*4^ | NA |  | 0.16 | ^*4^ |
| Genistein | Y | Y | 270.2 | 1.45 | 0.2 |  | 26.0 |  | 1.29  0.589 | ^*1^  ^*7^ | 0.0369 | ^*1^ | NA |  | 0.0238  0.181 | ^*1^  ^*7^ | 0.0128 | ^*1^ | NA |  |
| Indomethacin | Y | Y | 357.8 | 0.75 | 3.2 | ^*1^ | 12.0 | ^*1^ | 0.17 | ^*4^ | NA |  | 0.27 | ^*4^ | 0.110 | ^*4^ | NA |  | NA |  |
| Lamotrigine | Y | Y | 256.1 | 1.68 | 1.4 | ^*1^ | 1.2 | ^*1^ | 0.86 | ^*4^ | 0.586  0.875 | ^*1^  ^*9^ | 1.1 | ^*4^ | 0.88  1.40 | ^*4^  ^*3^ | 0.861  0.678 | ^*1^  ^*9^ | 2.80 | ^*3^ |
| Levofloxacin | Y | N | 361.4 | NA | NA |  | NA |  | 0.19 | ^*4^ | NA |  | 0.18 | ^*4^ | 0.120 | ^*4^ | NA |  | NA |  |
| Loperamide | Y | Y | 477.1 | 3.94 | 92 | ^*1^ | 0.97 | ^*1^ | 0.037  0.038 | ^*4^  ^*7^ | NA |  | NA |  | 0.024  0.007  0.00896 | ^*3^  ^*4^  ^*7^ | 0.04 | ^*8^ | NA |  |
| M3G | Y | N | NA | NA | NA |  | NA |  | 0.049 | ^*4^ | NA |  | 0.081 | ^*4^ | 0.011 | ^*4^ | NA |  | NA |  |
| M6G | Y | N | NA | NA | NA |  | NA |  | 0.017 | ^*4^ | NA |  | 0.1 | ^*4^ | 0.008 | ^*4^ | NA |  | NA |  |
| Methotrexate | Y | Y | 454.4 | -5.22 | 1.4 | ^*1^ | 1.2 | ^*1^ | 0.007 | ^*4^ | 0.089 | ^*1^ | 0.062 | ^*4^ | 0.006 | ^*4^ | 0.041 | ^*1^ | NA |  |
| Metoprolol | Y | Y | 267.4 | -0.25 | 7.0 | ^*1^ | 0.7 |  | 0.43 | ^*4^ | NA |  | 0.93 | ^*4^ | 0.640 | ^*4^ | NA |  | NA |  |
| Mirtazapine | N | Y | 265.4 | 2.40 | 0.9 | ^*1^ | 0.9 | ^*1^ | NA |  | NA |  | NA |  | 1.611 | ^*3^ | NA |  | 0.79 | ^*3^ |
| Morphine | Y | N | 285.3 | NA | NA |  | NA |  | 0.4 | ^*4^ | NA |  | 0.51 | ^*4^ | 0.150 | ^*4^ | NA |  | NA |  |
| Moxalactam | Y | N | 518.5 | -4.93 | NA |  | NA |  | 0.02 | ^*4^ | 0.137 | ^*1^ | 0.41 | ^*4^ | 0.019 | ^*4^ | NA |  | NA |  |
| Nadolol | Y | N | 309.4 | -0.86 | NA |  | NA |  | 0.041 | ^*4^ | NA |  | NA |  | 0.037 | ^*4^ | NA |  | NA |  |
| Nelfinavir | Y | Y | 567.8 | 5.68 | 296 | ^*1^ | 1.4 | ^*1^ | 0.067 | ^*4^ | 0.640 | ^*1^ | 0.045 | ^*4^ | 0.019 | ^*4^ | 0.008 | ^*1^ | NA |  |
| Nitrofurantoin | Y | N | 238.2 | NA | NA |  | NA |  | 0.0099 | ^*4^ | NA |  | NA |  | 0.011 | ^*4^ | NA |  | NA |  |
| Norfloxacin | Y | N | 319.3 | NA | NA |  | NA |  | 0.018 | ^*4^ | NA |  | 0.11 | ^*4^ | 0.028 | ^*4^ | NA |  | NA |  |
| Olanzapine | N | Y | 312.4 | 1.90 | 3.3 | ^*1^ | 0.8 | ^*1^ | NA |  | NA |  | NA |  | 2.450 | ^*3^ | NA |  | 0.15 | ^*3^ |
| Oxprenolol | Y | N | 265.4 | NA | NA |  | NA |  | 0.1 | ^*4^ | NA |  | NA |  | 0.200 | ^*4^ | NA |  | NA |  |
| Oxycodone | Y | N | 315.4 | NA | NA |  | NA |  | 0.65 | ^*4^ | NA |  | NA |  | 1.030 | ^*4^ | NA |  | NA |  |
| Oxymorphone | Y | N | 301.3 | NA | NA |  | NA |  | 0.91 | ^*4^ | NA |  | NA |  | 0.790 | ^*4^ | NA |  | NA |  |
| Pefloxacin | Y | Y | 333.4 | -2.42 | 9.9 | ^*1^ | 17.0 | ^*1^ | 0.389 | ^*7^ | 0.512 | ^*1^ | 0.743 | ^*12^ | 0.199 | ^*7^ | 0.452 | ^*1^ | NA |  |
| Phenytoin | Y | Y | 252.3 | 2.39 | 2.7 | ^*1^ | 1.0 | ^*1^ | 0.396 | ^*7^ | NA |  | NA |  | 0.316  0.447 | ^*3^  ^*7^ | NA |  | 0.59 | ^*3^ |
| Pindolol | Y | Y | 248.3 | -0.32 | 5.7 | ^*1^ | 1.2 | ^*1^ | 0.11 | ^*4^ | NA |  | 0.52 | ^*4^ | 0.500 | ^*4^ | NA |  | NA |  |
| Pitavastatin | Y | Y | 421.5 | 0.39 | 26 | ^*1^ | 10.0 | ^*1^ | NA |  | 0.129 | ^*1^ | NA |  | NA |  | 0.242 | ^*1^ | NA |  |
| Prazosin | Y | Y | 383.4 | 1.11 | 52 | ^*1^ | 18.0 | ^*1^ | NA |  | 0.289 | ^*1^ | NA |  | NA |  | 0.253 | ^*1^ | NA |  |
| Propranolol | Y | Y | 259.3 | 1.15 | 0.9 | ^*1^ | 1.0 |  | 0.49 | ^*4^ | 0.461 | ^*9^ | 0.42 | ^*4^ | 0.610 | ^*4^ | 0.947 | ^*9^ | NA |  |
| Quinidine | Y | Y | 324.4 | 1.73 | 173 | ^*1^ | 0.9 | ^*1^ | 0.419  0.0911 | ^*1^  ^*7^ | 0.197  0.169 | ^*1^  ^*9^ | NA |  | 0.0359  0.026 | ^*1^  ^*7^ | 0.0976  0.0744  0.0658 | ^*1^  ^*8^  ^*9^ | NA |  |
| Rifampicin | Y | N | 822.9 | NA | NA |  | NA |  | 0.34 | ^*4^ | NA |  | 2.2 | ^*4^ | 0.035 | ^*4^ | NA |  | NA |  |
| Rolipram | N | Y | 275.3 | 1.72 | 1.5 | ^*1^ | 1.1 | ^*1^ | NA |  | NA |  | NA |  | NA |  | NA |  | 1.00 | ^*3^ |
| Salicylic acid | Y | N | 138.1 | -0.77 | NA | ^*1^ | NA |  | 0.16 | ^*4^ | NA |  | 0.19 | ^*4^ | 0.190 | ^*4^ | NA |  | NA |  |
| Saquinavir | Y | N | 670.8 | NA | NA |  | NA |  | NA |  | NA |  | 0.096 | ^*4^ | 0.055 | ^*4^ | NA |  | NA |  |
| Sertraline | Y | Y | 306.2 | 3.14 | 0.5 | ^*1^ | 1.2 | ^*1^ | 0.832 | ^*7^ | NA |  | NA |  | 1.86  1.85 | ^*3^  ^*7^ | NA |  | 0.12 | ^*3^ |
| Sorafenib | Y | Y | 464.8 | 4.26 | 4.8 | ^*1^ | 33.0 | ^*1^ | 2.06 | ^*1^ | 0.0798 | ^*1^ | NA |  | 0.0228 | ^*1^ | 0.0199 | ^*1^ | NA |  |
| Tacrine | Y | Y | 198.3 | 0.71 | 1.0 | ^*1^ | 1.1 | ^*1^ | 0.67 | ^*4^ | NA |  | 0.74 | ^*4^ | 0.780 | ^*4^ | NA |  | NA |  |
| Thiopental | Y | N | 242.3 | NA | NA |  | NA |  | 1.09  0.599 | ^*4^  ^*7^ | NA |  | 0.75 | ^*4^ | 1.53  0.911 | ^*4^  ^*7^ | NA |  | NA |  |
| Thioridazine | Y | Y | 370.6 | 3.69 | 3.7 | ^*1^ | 0.5 | ^*1^ | 0.21 | ^*4^ | NA |  | 1.4 | ^*4^ | 1.425  0.45 | ^*3^  ^*4^ | NA |  | NA |  |
| Topiramate | Y | Y | 339.4 | 2.14 | 25 |  | 0.7 |  | 0.63 | ^*4^ | NA |  | 1 | ^*4^ | 0.330 | ^*4^ | NA |  | NA |  |
| Tramadol | Y | N | 263.4 | NA | NA |  | NA |  | 0.71 | ^*4^ | NA |  | 1.44 | ^*4^ | 1.460 | ^*4^ | NA |  | NA |  |
| Trazodone | N | Y | 371.9 | 2.53 | 0.6 |  | 0.9 |  | NA |  | NA |  | NA |  | 0.957 | ^*3^ | NA |  | 0.93 | ^*3^ |
| Venlafaxine | N | Y | 277.4 | 1.43 | 7.6 | ^*1^ | 0.7 | ^*1^ | NA |  | NA |  | NA |  | 1.074 | ^*3^ | NA |  | 0.86 | ^*3^ |
| Verapamil | Y | Y | 454.6 | 2.38 | 23 | ^*1^ | 0.8 | ^*1^ | 0.55  0.11  0.333 | ^*1^  ^*4^  ^*7^ | 0.238  0.183 | ^*1^  ^*9^ | 1.13 | ^*4^ | 0.090  0.053  0.0786 | ^*1^  ^*4^  ^*7^ | 0.621  0.154  0.0789 | ^*1^  ^*8^  ^*9^ | 0.24 | ^*6^ |
| Zidovudine | Y | Y | 267.2 | -0.10 | 28 | ^*1^ | 18.0 | ^*1^ | 0.18 | ^*4^ | NA |  | 1.04 | ^*4^ | 0.090 | ^*4^ | NA |  | NA |  |

NA: Not available.

Y and N indicate whether data was used or not, respectively, for the correlation analysis between K_p,uu,brain_ and K_p,uu,CSF_ described in figure type a (Figure 2) and the neuropharmacokinetic model-based prediction of K_p,uu,brain_ and K_p,uu,CSF_ described in figure type b (Figure 3 and 4).

^*1^: Data internally obtained.

^*2^, ^*3^, ^*4^, ^*5^, ^*6^, ^*7^, ^*8^, ^*9^, ^*10^, ^*11^ and ^*12^: Data from literature information disclosed by Post *et al.* (24), Summerfield *et al.* (25), Friden *et al.* (21), Bauer *et al.* (26), Liu *et al.* (17), Kodaira *et al.* (20), Uchida *et al.* (31), Nagaya *et al.* (19), Gatti *et al.* (28), Togashi *et al.* (29), and Wolff *et al.* (30), respectively. Data wherein only total concentrations in plasma, CSF, and brain were available, the total concentration ratio in plasma to CSF or brain (K_p,CSF_ or K_p,brain_, respectively) was estimated, followed by calculation of K_p,uu,CSF_ or K_p,uu,brain_ based on Eq. 4, 5, and 6 using the unbound fractions in the plasma and brain that were obtained internally.

**Supplemental Table 2 (Continued)**

| Compound name | Figure type | | Mw | LogD | Efflux ratio | | | | K_p,uu,CSF_ | | | | | | K_p,uu,brain_ | | | | | |
| --- | --- | --- | --- | --- | --- | --- | --- | --- | --- | --- | --- | --- | --- | --- | --- | --- | --- | --- | --- | --- |
|  | a | b |  |  | MDR1 | | BCRP | | Rat | | Monkey | | Human | | Rat | | Monkey | | Human | |
| TK-1 | Y | Y | 433.5 | 3.06 | 29.1 | ^*1^ | 0.8 | ^*1^ | 0.22 | ^*1^ | NA |  | NA |  | 0.080 | ^*1^ | NA |  | NA |  |
| TK-2 | Y | Y | 388.4 | 2.67 | 8.4 | ^*1^ | 3.5 | ^*1^ | 0.08 | ^*1^ | NA |  | NA |  | 0.19 | ^*1^ | NA |  | NA |  |
| TK-3 | Y | Y | 336.4 | 2.08 | 3.2 | ^*1^ | 2.8 | ^*1^ | 0.16 | ^*1^ | NA |  | NA |  | 0.060 | ^*1^ | NA |  | NA |  |
| TK-4 | Y | Y | 361.3 | 2.96 | 3.8 | ^*1^ | 1.0 | ^*1^ | 0.35 | ^*1^ | NA |  | NA |  | 0.15 | ^*1^ | NA |  | NA |  |
| TK-5 | Y | Y | 438.5 | 2.20 | 24.3 | ^*1^ | 1.3 | ^*1^ | 0.21 | ^*1^ | NA |  | NA |  | 0.070 | ^*1^ | NA |  | NA |  |
| TK-6 | Y | Y | 430.5 | 3.64 | 49.1 | ^*1^ | 3.1 | ^*1^ | 0.19 | ^*1^ | NA |  | NA |  | 0.07 | ^*1^ | NA |  | NA |  |
| TK-7 | Y | Y | 427.4 | 3.12 | 1.8 | ^*1^ | 0.8 | ^*1^ | 0.28 | ^*1^ | NA |  | NA |  | 0.23 | ^*1^ | NA |  | NA |  |
| TK-8 | Y | Y | 337.4 | 2.67 | 1.4 | ^*1^ | 1.7 | ^*1^ | 0.28 | ^*1^ | NA |  | NA |  | 0.05 | ^*1^ | NA |  | NA |  |
| TK-9 | Y | Y | 345.3 | 3.32 | 4.8 | ^*1^ | 1.7 | ^*1^ | 0.36 | ^*1^ | NA |  | NA |  | 0.21 | ^*1^ | NA |  | NA |  |
| TK-10 | Y | Y | 438.4 | 3.00 | 1.4 | ^*1^ | 0.8 | ^*1^ | 0.61 | ^*1^ | NA |  | NA |  | 0.14 | ^*1^ | NA |  | NA |  |
| TK-11 | Y | Y | 333.3 | 2.90 | 8.3 | ^*1^ | 1.6 | ^*1^ | 0.39 | ^*1^ | NA |  | NA |  | 0.2 | ^*1^ | NA |  | NA |  |
| TK-12 | Y | Y | 464.4 | 1.91 | 43.1 | ^*1^ | 0.8 | ^*1^ | 0.15 | ^*1^ | NA |  | NA |  | 0.02 | ^*1^ | NA |  | NA |  |
| TK-13 | Y | Y | 349.3 | 2.93 | 6.3 | ^*1^ | 1.3 | ^*1^ | 0.26 | ^*1^ | NA |  | NA |  | 0.12 | ^*1^ | NA |  | NA |  |
| TK-14 | Y | Y | 434.5 | 2.41 | 19.1 | ^*1^ | 0.8 | ^*1^ | 0.18 | ^*1^ | NA |  | NA |  | 0.04 | ^*1^ | NA |  | NA |  |
| TK-15 | Y | Y | 433.5 | 3.05 | 19.8 | ^*1^ | 1.4 | ^*1^ | 0.19 | ^*1^ | NA |  | NA |  | 0.08 | ^*1^ | NA |  | NA |  |
| TK-16 | Y | Y | 388.4 | 2.60 | 4.9 | ^*1^ | 1.9 | ^*1^ | 0.07 | ^*1^ | NA |  | NA |  | 0.03 | ^*1^ | NA |  | NA |  |
| TK-17 | Y | Y | 517.0 | 2.01 | 5.1 | ^*1^ | 2.0 | ^*1^ | 0.42 | ^*1^ | NA |  | NA |  | 0.02 | ^*1^ | NA |  | NA |  |
| TK-18 | Y | Y | 442.3 | 2.77 | 5.6 | ^*1^ | 2.4 | ^*1^ | 0.17 | ^*1^ | NA |  | NA |  | 0.16 | ^*1^ | NA |  | NA |  |
| TK-19 | Y | Y | 345.8 | 0.43 | 6.9 | ^*1^ | 2.4 | ^*1^ | 0.29 | ^*1^ | NA |  | NA |  | 0.31 | ^*1^ | NA |  | NA |  |
| TK-20 | Y | Y | 353.3 | 1.13 | 3.0 | ^*1^ | 17.0 | ^*1^ | 0.5 | ^*1^ | NA |  | NA |  | 0.04 | ^*1^ | NA |  | NA |  |
| TK-21 | Y | Y | 466.5 | 3.01 | 105.3 | ^*1^ | 2.2 | ^*1^ | NA |  | NA |  | NA |  | 0.02 | ^*1^ | NA |  | NA |  |
| TK-22 | Y | Y | 452.5 | 2.73 | 132.4 | ^*1^ | 8.2 | ^*1^ | 0.65 | ^*1^ | NA |  | NA |  | 0.02 | ^*1^ | NA |  | NA |  |
| TK-23 | Y | Y | 460.4 | 3.51 | 9.6 | ^*1^ | 3.1 | ^*1^ | NA |  | NA |  | NA |  | 0.454 | ^*1^ | NA |  | NA |  |
| TK-24 | Y | Y | 315.4 | 0.95 | 12.0 | ^*1^ | 1.7 | ^*1^ | NA |  | NA |  | NA |  | 0.378 | ^*1^ | NA |  | NA |  |
| TK-25 | Y | Y | 328.2 | 0.87 | 1.4 | ^*1^ | 1.7 | ^*1^ | NA |  | NA |  | NA |  | 0.403 | ^*1^ | NA |  | NA |  |
| TK-26 | Y | Y | 342.3 | 1.34 | 1.9 | ^*1^ | 1.2 | ^*1^ | NA |  | NA |  | NA |  | 0.46 | ^*1^ | NA |  | NA |  |
| TK-27 | Y | Y | 327.3 | 1.65 | 1.9 | ^*1^ | 1.3 | ^*1^ | NA |  | NA |  | NA |  | 0.358 | ^*1^ | NA |  | NA |  |
| TK-28 | Y | Y | 360.8 | 3.29 | 4.2 | ^*1^ | 1.0 | ^*1^ | NA |  | NA |  | NA |  | 0.356 | ^*1^ | NA |  | NA |  |
| TK-29 | Y | Y | 345.2 | 1.82 | 1.3 | ^*1^ | 0.9 | ^*1^ | NA |  | NA |  | NA |  | 0.483 | ^*1^ | NA |  | NA |  |
| TK-30 | Y | Y | 426.3 | 0.38 | 15.0 | ^*1^ | 14.0 | ^*1^ | NA |  | NA |  | NA |  | 0.054 | ^*1^ | NA |  | NA |  |
| TK-31 | Y | Y | 363.2 | 1.82 | 3.6 | ^*1^ | 3.4 | ^*1^ | NA |  | NA |  | NA |  | 0.175 | ^*1^ | NA |  | NA |  |
| TK-32 | Y | Y | 376.4 | 3.15 | 3.4 | ^*1^ | 0.8 | ^*1^ | NA |  | NA |  | NA |  | 0.412 | ^*1^ | NA |  | NA |  |
| TK-33 | Y | Y | 412.9 | 0.98 | 48.0 | ^*1^ | 22.0 | ^*1^ | NA |  | NA |  | NA |  | 0.035 | ^*1^ | NA |  | NA |  |
| TK-34 | Y | Y | 442.8 | 2.27 | 4.6 | ^*1^ | 28.0 | ^*1^ | NA |  | NA |  | NA |  | 0.011 | ^*1^ | NA |  | NA |  |
| TK-35 | Y | Y | 303.3 | 1.82 | 4.4 | ^*1^ | 1.4 | ^*1^ | NA |  | NA |  | NA |  | 0.175 | ^*1^ | NA |  | NA |  |
| TK-36 | Y | Y | 464.5 | 3.28 | 3.7 | ^*1^ | 1.1 | ^*1^ | NA |  | NA |  | NA |  | 0.149 | ^*1^ | NA |  | NA |  |
| TK-37 | Y | Y | 500.5 | 3.07 | 26.0 | ^*1^ | 1.0 | ^*1^ | NA |  | 0.444 | ^*1^ | NA |  | NA |  | 0.173 | ^*1^ | NA |  |
| TK-38 | Y | Y | 486.5 | 4.04 | 13.0 | ^*1^ | 1.0 | ^*1^ | NA |  | 0.502 | ^*1^ | NA |  | NA |  | 0.343 | ^*1^ | NA |  |
| TK-39 | Y | Y | 492.6 | 3.42 | 27.7 | ^*1^ | 1.0 | ^*1^ | NA |  | 0.901 | ^*1^ | NA |  | NA |  | 0.437 | ^*1^ | NA |  |
| TK-40 | Y | Y | 526.6 | 2.14 | 80.2 | ^*1^ | 1.1 | ^*1^ | NA |  | 0.298 | ^*1^ | NA |  | NA |  | 0.046 | ^*1^ | NA |  |
| TK-41 | Y | Y | 366.3 | 1.92 | 14.6 | ^*1^ | 2.2 | ^*1^ | NA |  | 0.733 | ^*1^ | NA |  | NA |  | 0.850 | ^*1^ | NA |  |
| TK-42 | Y | Y | 449.4 | 0.57 | 2.5 | ^*1^ | 8.0 | ^*1^ | NA |  | 0.533 | ^*1^ | NA |  | NA |  | 0.390 | ^*1^ | NA |  |
| TK-43 | Y | Y | 467.4 | 0.54 | 10.4 | ^*1^ | 2.8 | ^*1^ | NA |  | 0.548 | ^*1^ | NA |  | NA |  | 0.740 | ^*1^ | NA |  |

NA: Not available.

TK-1 to TK-43: Internal molecules.

Y and N indicate whether data was used or not, respectively, for the correlation analysis between K_p,uu,brain_ and K_p,uu,CSF_ described in figure type a (Figure 2) and the neuropharmacokinetic model-based prediction of K_p,uu,brain_ and K_p,uu,CSF_ described in figure type b (Figure 3 and 4).

^*1^: Data internally obtained.

**Supplemental Table 3　 Dose regimen for *in vivo* experiments using monkeys in this study**

|  |  |  |  |  |
| --- | --- | --- | --- | --- |
| Cassette dosing No. | Compound | Bolus dose  (mg/kg) | Infusion dose (mg/h/kg) | Vehicle solution |
| 1 | Carbamazepine | 0.220 | 0.100 | DMA-1,3-butanediol (1:1, v/v) |
| 1 | Dapsone | 0.140 | 0.0300 | DMA-1,3-butanediol (1:1, v/v) |
| 1 | Erlotinib | 0.0915 | 0.156 | DMA-1,3-butanediol (1:1, v/v) |
| 1 | Etoposide | 0.0200 | 0.0100 | DMA-1,3-butanediol (1:1, v/v) |
| 2 | Lamotrigine | 0.296 | 0.0160 | DMA-1,3-butanediol (1:1, v/v) |
| 2 | Pefloxacin | 0.0559 | 0.0559 | DMA-1,3-butanediol (1:1, v/v) |
| 2 | Methotrexate | 0.0880 | 0.0880 | DMA-1,3-butanediol (1:1, v/v) |
| 2 | Nelfinavir | 0.212 | 0.116 | DMA-1,3-butanediol (1:1, v/v) |
| 2 | Baclofen | 0.0400 | 0.144 | DMA-1,3-butanediol (1:1, v/v) |
| 3 | Atenolol | 0.0250 | 0.00500 | Saline |
| 3 | Moxalactam | 0.00184 | 0.000919 | Saline |
| 4 | Dantrolene | 0.200 | 0.200 | DMA-1,3-butanediol (1:1, v/v) |
| 4 | Daidzein | --- | 0.300 | DMA-1,3-butanediol (1:1, v/v) |
| 4 | Genistein | --- | 0.375 | DMA-1,3-butanediol (1:1, v/v) |
| 4 | Quinidine | 0.300 | 0.200 | DMA-1,3-butanediol (1:1, v/v) |
| 4 | Pitavastatin | --- | 0.400 | DMA-1,3-butanediol (1:1, v/v) |
| 4 | Prazosin | 0.200 | 0.300 | DMA-1,3-butanediol (1:1, v/v) |
| 4 | Sorafenib | 0.300 | 0.025 | DMA-1,3-butanediol (1:1, v/v) |
| 4 | Verapamil | 0.150 | 0.0500 | DMA-1,3-butanediol (1:1, v/v) |

---: Not administered

Dosing volume for bolus dose: 0.2 mL/kg in cassette dosing No. 1, 2, and 4 and 0.4 mL/kg in cassette dosing No. 3

Dosing volume for infusion dose: 0.25 mL/kg

**Supplemental Table 4　 LC/MS/MS parameters for quantification**

|  |  |  |  |  |  |  |
| --- | --- | --- | --- | --- | --- | --- |
| Compound | Q1 | Q3 | DP | EP | CE | CXP |
| Carbamazepine | 237.06 | 194.01 | 50 | 10 | 25 | 15 |
| Dapsone | 249.14 | 156.01 | 40 | 10 | 25 | 15 |
| Erlotinib | 394.19 | 277.93 | 60 | 10 | 45 | 15 |
| Etoposide | 589.20 | 299.11 | 120 | 10 | 25 | 15 |
| Lamotrigine | 255.83 | 210.85 | 60 | 10 | 35 | 15 |
| Pefloxacin | 334.10 | 233.09 | 50 | 10 | 35 | 15 |
| Methotrexate | 455.32 | 308.04 | 50 | 10 | 25 | 15 |
| Nelfinavir | 568.35 | 330.09 | 80 | 10 | 45 | 15 |
| Moxalactam | 521.22 | 377.20 | 51 | 10 | 19 | 12 |
| Atenolol | 267.18 | 190.09 | 40 | 10 | 25 | 15 |
| Baclofen | 214.10 | 151.02 | 30 | 10 | 25 | 15 |
| Dantrolene | 312.98 | 199.80 | -30 | -10 | -25 | -15 |
| Daidzein | 255.00 | 227.25 | 96 | 10 | 35 | 15 |
| Genistein | 271.03 | 153.01 | 96 | 10 | 35 | 15 |
| Quinidine | 325.23 | 172.07 | 96 | 10 | 55 | 15 |
| Pitavastatin | 422.16 | 274.09 | 112 | 10 | 65 | 15 |
| Prazosin | 384.10 | 247.10 | 91 | 10 | 39 | 24 |
| Sorafenib | 463.02 | 193.94 | -40 | -10 | -25 | -15 |
| Verapamil | 455.28 | 165.12 | 64 | 10 | 45 | 15 |
| Alprenolol | 296.10 | 214.20 | 51 | 10 | 47 | 15 |
| Dichlofenac | 214.10 | 151.02 | 30 | 10 | 25 | 15 |
